# Dynamics of HIV self-testing uptake among sexual and gender minorities: pre and during COVID-19

**DOI:** 10.11606/s1518-8787.2024058006011

**Published:** 2024-10-11

**Authors:** Laio Magno, Dulce Ferraz, Eliana Miura Zucchi, Jony Arrais Junior Pinto, Fabiane Soares, Alexandre Grangeiro, Dirceu Greco, Ines Dourado

**Affiliations:** I Universidade do Estado da Bahia. Departamento de Ciências da Vida. Salvador, BA, Brasil Universidade do Estado da Bahia Departamento de Ciências da Vida BA Salvador Brasil; II Universidade Federal da Bahia. Instituto de Saúde Coletiva. Salvador, BA, Brasil Universidade Federal da Bahia Instituto de Saúde Coletiva BA Salvador Brasil; III Fundação Oswaldo Cruz. Escola Fiocruz de Governo. Brasília, DF, Brasil Fundação Oswaldo Cruz Escola Fiocruz de Governo DF Brasília Brasil; IV Universidade Católica de Santos. Programa de Pós-Graduação em Saúde Coletiva. Santos, SP, Brasil Universidade Católica de Santos Programa de Pós-Graduação em Saúde Coletiva SP Santos Brasil; V Universidade Federal Fluminense. Departamento de Estatística. Niterói, RJ, Brasil Universidade Federal Fluminense Departamento de Estatística RJ Niterói Brasil; VI Universidade de São Paulo. Faculdade de Medicina. São Paulo, SP, Brasil Universidade de São Paulo Faculdade de Medicina SP São Paulo Brasil; VII Universidade Federal de Minas Gerais. Faculdade de Medicina. Belo Horizonte, MG, Brasil Universidade Federal de Minas Gerais Faculdade de Medicina MG Belo Horizonte Brasil

**Keywords:** HIV Self-testing, Adolescents, Men Who Have Sex With Men, Transgender Women, COVID-19, Brazil

## Abstract

**OBJECTIVE:** To identify the factors associated with HIV self-testing (HIVST) uptake among adolescent men who have sex with men (AMSM) and adolescent transgender women (ATGW) before and during the COVID-19 pandemic. **METHODS:** A cross-sectional HIVST uptake study was conducted among AMSM and ATGW. Peer educators and health professionals began providing HIVST in February 2019. The outcome was the HIVST uptake before and during the COVID-19 pandemic. The association between each predictor and outcome in each period was analyzed using simple and multiple logistic regressions, estimating odds ratios, and their respective 95% confidence intervals. **RESULTS:** The uptake was 229/510 (44.9%) and 382/1,075 (35.5%) before and during the pandemic. During the pre-pandemic period, HIVST uptake was higher in participants who reported receptive anal sex. During the pandemic, uptake was lower in participants with a steady sexual partner and higher in those with frequent oral sex with a steady partner in the previous three months. Before and during the pandemic, HIVST uptake was lower in ATGW and higher in those aged 18–19 years and in participants who lived alone. **CONCLUSIONS:** Uptake decreased during the pandemic. Sexual behavioral factors associated with HIVST uptake changed during the COVID-19 pandemic, showing the fluid dynamics of sexuality in AMSM and ATGW during this period. HIV programs can optimize the implementation of HIVST among adolescents and young people by incorporating effective and differentiated service delivery models to increase HIV testing uptake and to reach undiagnosed individuals effectively.

## INTRODUCTION

HIV self-testing (HIVST) is an alternative method to reach populations that have difficulty accessing testing, such as men who have sex with men (MSM) and transgender women (TGW) ^2023^ . Specifically, studies on MSM in several countries have shown high HIVST acceptability ^2018^^,^^2019^ , increased testing frequency, and an association between the use of HIVST, subsequent facility-based testing, and consistent condom use ^2019^ . Reports of risk compensation (e.g., an increased number of partners and decreased condom use) and adverse psychological effects associated with HIVST are less common ^2017^^,^^2017^ .

Previous studies have found that adolescents at increased HIV risk show a high intention to use HIVST but doubt the test characteristics and form of provision. Data from the formative phase of the HIV Self-Testing Africa Initiative (STAR) study, conducted in Zimbabwe and Zambia, showed that some young people doubted the accuracy of different self-tests. Additionally, confidentiality, secrecy, and the desire for autonomy influenced young people’s attitudes toward facility-based and self-test acceptance and use ^2017^ . Among Brazilian adolescent MSM (AMSM) and TGW (ATGW), the HIVST offered several perceived advantages, including speed, privacy, autonomy, and the ability to manage emotional well-being and stigma. However, concerns about managing a positive result, fear of HIVST via digital puncture, and the effectiveness of this method as a prevention strategy remain ^2023^ .

The COVID-19 pandemic, which has negatively affected HIV service delivery in several countries, has increased the importance of HIVST and the interest in its use ^2021^^,^^2021^ , partly because of physical distancing measures, particularly in adolescents and young people from sexual and gender minorities ^2022^ . This was particularly marked in Brazil, one of the most affected countries by the pandemic, which experienced difficulties maintaining HIV prevention, testing, and treatment services before COVID-19 vaccination became available ^2020^ .

In response to the COVID-19 pandemic in 2020, the Brazilian Ministry of Health implemented large-scale HIV self-testing (HIVST) ^2020a^^,^^2020b^ , aligning with the World Health Organization’s (WHO) recommendation in 2016 ^2019^ . This initiative aimed to alleviate healthcare wait times, reduce overcrowding ^2020a^ , and enhance access to timely diagnosis ^2020b^ . Nonetheless, there remains a limited understanding of the use of HIVST in the Brazilian context before and during the COVID-19 pandemic, especially among adolescents. Furthermore, to our knowledge, there are no studies with adolescents from key populations that have investigated which characteristics are associated with HIVST uptake before and during the pandemic period. Thus, this study aims to identify the factors associated with HIVST uptake in AMSM and ATGW in three Brazilian cities before and during the COVID-19 pandemic.

## METHODS

### Study Design, Population, and Recruitment

A cross-sectional HIVST uptake study nested within the PrEP1519 cohort, which was the first in Latin America to analyze daily oral pre-exposure prophylaxis (PrEP) in AMSM and ATGW aged 15–19 years. This study included three cities in Brazil: Salvador, São Paulo, and Belo Horizonte. The primary objective of the PrEP1519 study was to estimate the effectiveness of daily oral PrEP use in AMSM and ATGW aged 15–19 years at high risk of HIV infection, and one of the secondary objectives was to evaluate the distribution and use of HIVST in these populations ^2022^ .

The study population comprised adolescents aged 15–19 years, enrolled in the study between February 2019 and March 2021, self-declared as AMSM or ATGW, living, working, or attending social spaces in one of the three studied cities, and who reported sexual intercourse that increased the risk of HIV infection at least once in their lives.

The demand creation strategies (DCS) developed for the study included in-person (i.e., peer recruitment, referrals by health services and non-governmental organizations [NGOs]) and online approaches (i.e., peer educators recruiting via hook-up applications and other social media). All adolescents who accessed the DCS received information about the study, and those who agreed to participate provided written informed consent ^2022^ . Those assessed as being at risk of HIV infection were invited to enroll at a PrEP clinic, where they self-selected to participate in one of the two arms of the demonstration project: i) the PrEP arm included those willing to enroll in daily use of oral PrEP with a tenofovir and emtricitabine (TDF/FTC) combination, and ii) the non-PrEP arm included those who were eligible for PrEP but chose not to use it and, instead, opted to receive other HIV combination prevention methods (i.e., counseling, condoms, lubricant, and HIVST) ^2022^ .

### HIVST provision before and during the COVID-19 pandemic

HIVST kits (with a test, material information, condoms, and intimate lubricant) were provided by peer educators and health professionals along with prevention interventions developed in the context of DCS. These health education and prevention interventions were provided face-to-face in social places (e.g., parties and beaches) and virtually via dating apps and social networks (e.g., Grindr, Instagram, and Facebook etc.). HIVST kits were provided to all participants of interest. HIVST kits were provided during face-to-face interventions and by mail for virtual interventions. HIVST kits were also available upon request to participants already enrolled in the cohort during study appointments with health professionals. Participants could also request up to five HIVST kits for use or contact at any time during the study period. After the onset of the pandemic, HIVST kits were sent more frequently to participants using PrEP.

When the HIVST kits were provided, participants were informed about the lower effectiveness of HIVST as a prevention strategy compared to the regular use of condoms and PrEP. In addition, printed information and Internet videos on performing the self-test were provided (e.g., YouTube video: https://www.youtube.com/watch?v=iYCuQ09Cu6w&t=9s ). In-person interventions only occurred before restrictive measures against the COVID-19 pandemic were introduced. By contrast, virtual interventions were provided throughout the study and intensified when physical distancing measures were implemented.

### Data Collection Instruments

When the HIVST kits were distributed, a questionnaire was administered to collect the following information: testing motivation, target person of the test (i.e., the participant, partner, and/or friend), the number of tests requested, and how they were provided (i.e., mail, in-service pickup, or provided in-service). In addition, those included in the PrEP1519 study cohort completed a socio-behavioral questionnaire during enrolment.

### Adaptation of Services during the COVID-19 Pandemic

In 2020, all the cities participating in the PrEP1519 study implemented quarantine and social isolation measures. This study continued to provide services and adapt to the new situation by strengthening its social media and telehealth infrastructure. Thus, it includes a telehealth option and simplified PrEP initiation and retention procedures, DCS, HIVST delivery, and peer-to-peer browsing using smartphone text messaging and online social media ^2020^ .

### Study Variables

The study period was divided into two discrete periods: before the COVID-19 pandemic (February 21, 2019, to March 15, 2020) and during the pandemic (March 16, 2020, to March 31, 2021). The study outcome was HIVST uptake (yes, no). Uptake was defined as a participant requesting at least one HIVST kit during the study period.

In addition, the association of other factors was investigated. The variables were chosen considering three dimensions: sociodemographic, sexual behavior, and violence. Therefore, we decided to investigate the characteristics of participants who requested HIVST before and after the COVID-19 pandemic, and whether they differed or not.

The following variables were analyzed: i) sociodemographic: study population (AMSM, ATGW); age (15–17 years, 18–19 years); race/skin color (White, Black or Mixed-race, and Yellow, Indigenous or others), level of education (primary, secondary, and higher education); current living situation (with parents or other family members, with other people, alone); access to technology (high or low, defined by having a cell phone, computer, Internet access, car, and a maid), and being a member of, or having contact with a lesbian, gay, bisexual, transgender, queer, and intersex (LGBTQI+) group, social movement, or NGO (no or yes); ii) sexual behavior: steady sexual partner in the previous 3 months (no or yes), condom use in receptive anal sex with a steady partner in the previous 3 months (no or yes), casual sexual partner in the previous 3 months (no or yes), condom use in receptive anal sex with a casual partner in the previous 3 months (no or yes), receptive anal sex with a steady or casual partner in the previous 3 months (no or yes), frequent oral sex with a steady partner in the previous 3 months (no or yes), group sex in the previous 3 months (no or yes), transactional sex in the previous 3 months (no or yes); and iii) violence: sexual violence in the previous 6 months (no or yes).

Sociodemographic variables were constructed based on an analysis of the baseline database. Behavioral, violence, and sex work variables were based on all participant appointments during the period of interest (i.e., before or during the COVID-19 pandemic).

### Statistical Analysis

The variables in the HIVST provision questionnaire were descriptively reported as absolute frequencies and proportions. Subsequently, we analyzed, for two periods (i.e., before and during the COVID-19 pandemic), the predictors of HIVST uptake in participants who were enrolled in the PrEP1519 study (in either the PrEP or non-PrEP arm) and answered a socio-behavioral questionnaire. The analyses of the predictors in each period were conducted independently. Chi-square or Fisher’s exact tests were used to assess the statistical significance of differences in predictors between groups in each period. The association between each predictor variable and HIVST uptake in each period was analyzed using simple and multiple logistic regressions. To develop a multivariate logistic regression model, the model was initially adjusted for all variables (p-values < 0.10 in the simple model, and variables that were not statistically significant in the multivariate model were removed). Age and sex identity were included as adjustment variables in the final multivariate model. Odds ratios (OR) and their respective 95% confidence intervals (95%CI) were reported. The Hosmer-Lemeshow test was used to assess the model’s goodness of fit. The R software version 4.1.0 (R Foundation for Statistical Computing, Vienna, Austria) was used for all analyses.

### Ethical Aspects

This study was conducted following the Brazilian and international research ethics guidelines. This study was approved by the Research Ethics Committee (REC) of the World Health Organization (Protocol ID: Fiotec-PrEP Adolescent Study), Universidade Federal da Bahia (#3,224,384), Universidade de São Paulo (#3,082,360), and Universidade Federal de Minas Gerais (#3,303,594). Adolescents aged 18 and 19 agreed to participate and signed an informed consent form. For adolescents aged < 18 years, each participating city followed a different protocol. According to local court decisions, a parent or guardian signed an informed consent form and/or assent form signed by the adolescent.

## RESULTS

This study included 510 participants before the COVID-19 pandemic and 1,075 during the pandemic. The participants’ characteristics were similar in both periods. In the periods before and during the pandemic, most participants were AMSM (88.2% and 88.3%, respectively), aged 18–19 years (80.6% and 81.6%, respectively), of Black skin color or of Mixed-race (68.2% and 68.5%, respectively), with secondary education (60.4% and 66.2%, respectively), and living with parents or other family members (80.2% and 80.5%, respectively). Regarding sexual behavior, in the period before and during the pandemic, most reported not having a steady (60.0% and 67.6%, respectively) or casual sexual partner (60.0% and 55%, respectively) in the previous 3 months, inconsistent condom use in receptive anal sex with steady (75.0% and 80.2%, respectively) and casual (60.5% and 68.2%, respectively) sexual partners in the previous 3 months, receptive anal sex with a steady or casual partner in the previous 3 months (66.7% and 54.4%, respectively), frequent oral sex with a steady partner in the previous 3 months (93.3% and 95.6%, respectively), and not having group sex in the previous 3 months (82.0% and 84.6%, respectively). In the periods before and during the pandemic, a minority of participants reported transactional sex (3.5% and 3.8%, respectively) and sexual violence in the previous six months (9.7% and 11.0%, respectively). The only characteristic that differed between the two groups was access to technology, with 67.0% of the participants classified as having low access before the pandemic and 64.9% classified as having high access during the pandemic ( Table 1 ).

**Table 1.** Characteristics of study participants, PrEP1519 study, 2019–2021, Brazil.

**Characteristic**	**Pre-pandemic** ^a^ **n = 510**	**During the pandemic** ^b^ **n = 1,075**
	**n (%)**	**n (%)**
HIVST uptake		
Yes	229 (44.90)	382 (35.53)
No	281 (55.10)	693 (64.47)
Sociodemographic		
Population		
AMSM	448 (88.19)	947 (88.34)
ATGW	60 (11.81)	125 (11.66)
Age		
15–17 years	90 (19.40)	178 (18.46)
18–19 years	374 (80.60)	786 (81.54)
Race/skin color		
White	147 (28.82)	303 (28.19)
Black or Mixed-Race	348 (68.24)	736 (68.47)
Yellow, Indigenous, or other	15 (2.94)	36 (3.35)
Education		
Primary education	48 (10.32)	86 (8.93)
Secondary education	281 (60.43)	637 (66.15)
Higher education	136 (29.25)	240 (24.92)
Currently living situation		
With parents or other family members	373 (80.22)	776 (80.50)
With other people	62 (13.33)	129 (13.38)
Alone	30 (6.45)	59 (6.12)
Access to technology		
High	153 (32.97)	622 (64.93)
Low	311 (67.03)	336 (35.07)
Attended an event or member of an LGBTQI+ organized group, social movement, or NGO			
No	397 (85.56)	848 (88.06)
Yes	67 (14.44)	115 (11.94)
Sexual behavior		
Steady sexual partner in the previous 3 months		
No	279 (60.00)	579 (67.64)
Yes	186 (40.00)	277 (32.36)
Condom use in receptive anal sex with a steady partner in the previous 3 months		
Consistent use	60 (25.00)	83 (19.81)
Inconsistent use	180 (75.00)	336 (80.19)
Casual sexual partner in the previous 3 months		
No	279 (60.00)	468 (54.99)
Yes	186 (40.00)	383 (45.01)
Condom use in receptive anal sex with a casual partner in the previous 3 months		
Consistent use	119 (39.53)	164 (31.78)
Inconsistent use	182 (60.47)	352 (68.22)
Receptive anal sex with a steady or casual partner in the previous 3 months		
No partners or receptive anal sex	155 (33.33)	390 (45.56)
Receptive anal sex	310 (66.67)	466 (54.44)
Frequent oral sex with steady partner in the previous 3 months		
No	20 (6.73)	22 (4.41)
Yes	277 (93.27)	477 (95.59)
Group sex in the previous 3 months		
No	306 (82.04)	524 (84.65)
Yes	67 (17.96)	95 (15.35)
Transactional sex in the previous 3 months		
No	218 (96.46)	557 (96.20)
Yes	8 (3.54)	22 (3.80)
Violence		
Sexual violence in the previous 6 months		
No	307 (90.29)	138 (89.03)
Yes	33 (9.71)	17 (10.97)

HIVST: HIV self-testing; AMSM: adolescent men who have sex with men; LGBTQI+: lesbian, gay, bisexual, transgender, queer/questioning, and intersex; NGO: non-governmental organization; ATGW: adolescent transgender women.

a February 21, 2019, to March 15, 2020.

b March 16, 2020, to March 31, 2021.

A total of 491 participants received at least one HIVST kit during the study. The mean number of test requests before the pandemic was 1.8 (SD = 0.92), and during the pandemic, 1.7 (SD = 0.97). Before and during the COVID-19 pandemic, HIVST uptake was 229/510 (44.9%) and 382/1,075 (35.5%). The HIVST recipients mainly were friends and sexual partners before the COVID-19 pandemic (67.6% and 56.2%, respectively), and the participants themselves during the pandemic (82.9%) ( Table 2 ).

**Table 2.** HIVST kit requests, provision, and the target person before and during the COVID-19 pandemic, PrEP1519 study, 2019–2021, Brazil.

	**Overall** **(n = 1,585)**	**Pre-pandemic** ^a^ **(n = 510)**	**During the pandemic** ^b^ **(n = 1,075)**
	**n (%)**	**n (%)**	**n (%)**
Total number of participants who received a HIVST kit at least once	491	229	382
Total HIVST kits provided	1,290	570 (44.2)	720 (55.8)
HIVST target person			
Self	299 (36.8)	51 (17.1)	248 (82.9)
Sexual partner	153 (18.8)	86 (56.2)	67 (43.8)
Friend or other	253 (31.2)	171 (67.6)	82 (32.4)
Not registered	107 (13.2)	0 (0.0)	107 (100.0)

HIVST: HIV self-testing.

a February 21, 2019, to March 15, 2020.

b March 16, 2020, to March 31, 2021.

Before the COVID-19 pandemic, HIVST uptake was higher in participants with receptive anal sex with a steady or casual partner (OR _a_ = 1.53, 95%CI: 1.02–2.29). In contrast, during the COVID-19 pandemic, HIVST uptake was lower in participants with a steady sexual partner in the previous three months (OR _a_ = 0.57, 95%CI: 0.39–0.82) and higher in the age group 18–19 years (OR _a_ = 1.81, 95%CI:1.10–3.05, respectively), and in those who reported frequent oral sex with a steady partner in the previous three months (OR _a_ = 2.84, 95%CI: 1.08–8.85) ( Table 3 ).

Both before and during the pandemic, HIVST uptake was significantly higher in participants who lived alone (OR _a_ = 3.36, 95%CI: 1.50–8.29; and OR _a_ = 2.35, 95%CI: 1.01–5.78, respectively); and lower in ATGW (OR _a_ = 0.86, 95%CI: 0.46–1.59; and OR _a_ = 0.66, 95%CI: 0.34–1.23, respectively), although these results were not statistically significant ( Table 3 ).

**Table 3.** Factors associated with requesting HIVST before and during the COVID-19 pandemic, PrEP1519 study, 2019–2021, Brazil.

**Variable**	**Pre-pandemic** ^a^ **(n = 510)**				**During the pandemic** ^b^ **(n = 1,075)**			
	**HIVST uptake** **(n = 229)** **n (%)**	**p-value**	**Crude OR (95%CI)**	**Adjusted OR (95%CI)**	**HIVST uptake** **(n = 382)** **n (%)**	**p-value**	**Crude OR (95%CI)**	**Adjusted OR (95%CI)**
Sociodemographic		0.4951				0.5519		
Study population		0.7723				0.1339		
MSM	203 (88.65)		1.00 (ref.)	1.00 (ref.)	345 (90.31)		1.00 (ref.)	1.00 (ref.)
TGW	26 (11.35)		0.92 (0.532–1.585)	0.86 (0.463–1.586)	37 (9.69)		0.73 (0.484–1.093)	0.66 (0.338–1.231)
Age		0.9887				0.5519		
15–17 years	43 (19.37)		1.00 (ref.)	1.00 (ref.)	58 (15.85)		1.00 (ref.)	1.00 (ref.)
18–19 years	179 (80.63)		1.00 (0.633–1.594)	0.99 (0.618–1.603)	308 (84.15)		1.33 (0.948–1.891)	1.81 (1.100–3.045)
Race/skin color		0.4349				0.3826		
White	60 (26.20)		1.00 (ref.)		99 (25.92)		1.00 (ref.)	
Black or Mixed-race	163 (71.18)		1.28 (0.866–1.893)		268 (70.16)		1.18 (0.891–1.570)	
Yellow, Indigenous, or other	6 (2.62)		0.97 (0.310–2.823)		15 (3.93)		1.47 (0.716–2.962)	
Level of education		0.3141				0.2068		
Primary education	20 (8.97)		1.00 (ref.)		25 (6.85)		1.00 (ref.)	
Secondary education	131 (58.74)		1.22 (0.661–2.298)		246 (67.40)		1.54 (0.949–2.550)	
Higher education	72 (32.29)		1.58 (0.813–3.094)		94 (25.75)		1.57 (0.931–2.711)	
Current living situation		0.01572				0.1578		
Parents or other family members	173 (77.58)		1.00 (ref.)	1.00 (ref.)	292 (79.78)		1.00 (ref.)	1.00 (ref.)
Other people	28 (12.56)		0.95 (0.552–1.632)	1.00 (0.565–1.767)	45 (12.30)		0.89 (0.597–1.305)	0.92 (0.544–1.542)
Alone	22 (9.87)		3.18 (1.433–7.777)	3.36 (1.496–8.292)	29 (7.92)		1.60 (0.940–2.729)	2.35 (1.013–5.780)
Access to technology		0.0386				0.0903		
High	84 (37.67)		1.00 (ref.)		65 (37.79)		1.00 (ref.)	
Low	139 (62.33)		0.66 (0.449–0.979)		107 (62.21)		0.78 (0.594–1.033)	
Being a member of, or having contact with a LGBTQI+ group, social movement, or NGO		0.1093				0.4692		
No	196 (88.29)		1.00 (ref.)		317 (87.09)		1.00 (ref.)	
Yes	26 (11.71)		0.65 (0.379–1.098)		47 (12.91)		1.16 (0.775–1.717)	
Sexual behavior								
Steady sexual partner in the previous 3 months		0.1394				0.0644		
No	126 (56.50)		1.00 (ref.)		256 (71.11)		1.00 (ref.)	1.00 (ref.)
Yes	97 (43.50)		1.32 (0.913–1.922)		104 (28.89)		0.76 (0.565–1.016)	0.57 (0.394–0.823)
Condom use in receptive anal sex with steady partner in the previous 3 months		0.1560				0.3705		
Consistent use	27 (21.26)		1.00 (ref.)		34 (17.89)		1.00 (ref.)	
Inconsistent use	100 (78.74)		1.53 (0.851–2.764)		156 (82.11)		1.25 (0.770–2.045)	
Casual sexual partner in the previous 3 months		0.1394				0.4010		
No	126 (56.50)		1.00 (ref.)		204 (56.67)		1.00 (ref.)	
Yes	97 (43.50)		1.32 (0.913–1.922)		156 (43.33)		0.89 (0.676–1.169)	
Condom use in receptive anal sex with casual partner in the previous 3 months		0.5168				0.2041		
Consistent use	66 (41.25)		1.00 (ref.)		68 (28.94)		1.00 (ref.)	
Inconsistent use	94 (58.75)		0.86 (0.538–1.363)		167 (71.06)		1.27 (0.878–1.857)	
Receptive anal sex with a steady or casual partner in the previous 3 months		0.0256				0.6747		
No partners or no receptive anal sex	63 (28.25)		1.00 (ref.)	1.00 (ref.)	161 (44.72)		1.00 (ref.)	
Receptive anal sex	160 (71.75)		1.56 (1.056–2.308)	1.53 (1.021–2.294)	199 (55.28)		1.06 (0.807–1.393)	
Frequent oral sex with steady partner in the previous 3 months		0.9379				0.04079		
No	10 (6.85)		1.00 (ref.)		5 (2.28)		1.00 (ref.)	1.00 (ref.)
Yes	136 (93.15)		0.96 (0.384–2.421)		214 (97.72)		2.77 (1.074–8.526)	2.84 (1.081–8.857)
Group sex in the previous 3 months		0.05714				0.8275		
No	148 (78.31)		1.00 (ref.)		238 (85.00)		1.00 (ref.)	
Yes	41 (21.69)		1.68 (0.986–2.917)		42 (15.00)		0.95 (0.611–1.476)	
Transactional sex in the previous 3 months		0.4712 ^a^				0.8321		
No	95 (95.00)		1.00 (ref.)		266 (96.38)		1.00 (ref.)	
Yes	5 (5.00)		2.16 (0.517–10.732)		10 (3.62)		0.91 (0.379–2.148)	
Violence								
Sexual violence in the previous 6 months		0.2378				0.2757		
No	147 (91.30)		1.00 (ref.)		234 (98.32)		1.00 (ref.)	
Yes	14 (8.70)		0.80 (0.382–1.649)		4 (1.68)		0.54 (0.150–1.540)	

OR: odds ratio; 95%CI: 95% confidence interval; LGBTQI+: lesbian, gay, bisexual, transgender, queer/questioning, and intersex; NGO: non-governmental organization; ref: reference group; HIVST: HIV self-testing; MSM: men who have sex with men; TGW: transgender women.

a February 21, 2019, to March 15, 2020.

b March 16, 2020, to March 31, 2021.

## DISCUSSION

There were no differences in the participants’ sociodemographic characteristics before and during the COVID-19 pandemic, except for access to technology, which was higher during the pandemic.

The COVID-19 pandemic may have changed the motivation for HIVST: before the pandemic, the main motivation of participants was to provide HIVST kits to friends and partners; during the pandemic, they were more likely to request it for their own use. The pandemic may have interrupted the expansion of HIVST provisions in adolescent sexual and social networks. Conversely, within the scope of the PrEP1519 project, during periods of social distancing and strict sanitary measures against COVID-19, HIVST kits were mailed to participants as a follow-up with their PrEP pills ^2020^ , which may also explain this phenomenon. Therefore, comparisons between uptake before and after the pandemic may be compromised as supply conditions differ. Although the participants could access the service during the pandemic, the ease of receiving care at home, with telehealth, may have facilitated uptake.

The HIVST uptake during the two study periods indicates a moderate acceptance of this HIV testing method among adolescents. During the pre-pandemic period, when there were no social restrictions, almost half of the participants requested an HIVST kit for themselves or their networks of partners and friends. In addition, nearly one-third of the participants requested an HIVST kit during the pandemic, with the main purpose being self-care, which is consistent with the trend in adult Brazilian MSM and TGW during the pandemic ^2021^ . This result shows that, during a health crisis, the expansion of HIVST in PrEP services can help expand the use of prophylaxis in key populations by facilitating clinical follow-up and increasing adherence and retention in care compared with standard-of-care PrEP delivery ^2022^ .

Changes in partnerships and sexual practice factors associated with HIVST uptake before and during the pandemic may reinforce the assumption that self-testing availability is combined with incentives for greater risk management and self-care. Thus, participants in stable relationships during the COVID-19 pandemic were less likely to request an HIVST kit. Whereas, in the period before the pandemic, participants who engaged in anal sex were more likely to request HIVST and more likely to distribute HIVST kits to their sexual partners.

Additionally, a qualitative study of adolescents conducted during the pandemic showed that confinement resulted in a preponderance of single-partner relationships and a lower perception of risk, which may have resulted in a lower perception of the need for HIV testing ^2023^ . This lower perceived need for HIV testing is concerning; people living with HIV who are unaware of their serological status do not have access to timely treatment. This highlights the importance of measures to enable responsiveness to care needs, which may vary depending on the context of sexual interactions, and timely access to preventive inputs, especially with telehealth support.

Regarding the life contexts of young people and adolescents, greater autonomy of the family favors HIVST uptake. The PrEP1519 study also found that living alone favored the continuity of PrEP use and follow-up during the pandemic ^2023^ . During both study periods, participants living alone were more likely to request an HIVST, as they received the test kit with more privacy and were able to use it at convenient times and places. Compared with 15–17-year-old participants, the greater request for HIVST by 18–19-year-old participants during the pandemic may reflect greater autonomy with possibly greater privacy as well as a process of continuous care that has been learned and consolidated over time. Conversely, worse socioeconomic conditions have the opposite effect: no or precarious access to technology (such as the internet and mobile phones) may restrict the search for preventive care, probably due to less access to these technologies and less privacy to receive and use self-testing at home.

Considering the importance of the HIV epidemic among AMSM and ATGW in Brazil, strategies to facilitate HIVST uptake are important for these groups. Studies in Brazil and other countries have shown that this method is highly acceptable to young people ^2017^^-^^2015^ . Factors that increase the acceptability of this testing method include greater autonomy and confidentiality associated with its use and lower transportation costs to receive testing ^2017^ . Among young people, the cost is a determining factor in the acceptability of a method ^2017^ . This reinforces the importance of the HIVST in the *Sistema Único de Saúde* (SUS *–* Brazilian Unified Health System), which is Brazil’s publicly funded healthcare system. However, there is concern about how to manage positive HIVST results ^2018^ . Thus, it is important to ensure the option of being guided by health professionals during the test or when reading the results, as necessary and on request, which was offered during the PrEP1519 project.

Although the lower HIVST uptake in the ATGW group was not statistically significant in this study, it is likely to reflect the lower participation of this population in studies on combined prevention. Previous studies have shown a high acceptance of HIVST in adult TGW ^2016^^,^^2020^ . A mixed-methods study conducted in San Francisco, United States, showed that TGW were willing to use HIVST and preferred it over clinical-based testing, finding it easy to use and recommending it to others. Cost is also an associated factor when using this method ^2016^ . A literature review on factors that may influence the use and dissemination of HIVST found that TGW finds HIVST convenient and generally prefer this method ^2022^ due to its high confidentiality and possibility of being used at home. In contrast, potential barriers to the use among TGW include marginalization and stigma in health services, lack of acceptance of sexual partners, and fear of being stigmatized as persons living with HIV.

This study had some important limitations. First, the data analyzed were cross-sectional, and it is impossible to access a temporal relation between the predictors of HIVST uptake. In addition, this observational study included a non-probabilistic sample of volunteers with different economic profiles in the two study periods, which may have led to a selection bias in the comparisons between groups. Despite these limitations, this is an important study for understanding HIVST uptake in AMSM and ATGW during periods of health crises, such as the COVID-19 pandemic.

## CONCLUSIONS

To our knowledge, this is the first study to investigate HIVST uptake among AMSM and ATGW. This study confirms the appropriateness and feasibility of providing HIVST to adolescents at high risk of HIV infection and has shown its effectiveness even in lockdown situations. In addition, it was conducted during the COVID-19 pandemic, a period of crisis during which people could not freely attend health services to be tested for HIV. This study showed high HIVST uptake before the pandemic, and the main target group for the kits was the participants’ friends and partners. However, uptake decreased during the pandemic, with a shift toward greater use of self-testing by the participants themselves. In addition, this study showed that older age and living alone were associated with increased uptake. ATGW had a lower uptake, highlighting the need to target ATGW. Finally, it also showed that the COVID-19 pandemic changed the sexual behavioral factors associated with HIVST uptake, revealing the fluid dynamics of the sexuality of AMSM and ATGW.

HIV programs can optimize the implementation of HIVST among adolescents and young people, incorporating effective and differentiated service delivery models, linkage to antiretroviral therapy, PrEP, and support tools. Various service delivery options—such as community-based, facility-based, and online platforms—and distribution models—such as peer-to-peer and mail delivery—could effectively increase testing uptake and reach undiagnosed individuals. Social networks, mobile outreach, and secondary distribution are crucial for expanding reach to gender and sexual minority adolescents, particularly those in socially vulnerable groups.
